# Supplementary material for: Mental Health and Traumatization of Newly Arrived Asylum Seeker Adults in Finland: A Population-Based Study
Source: Int J Environ Res Public Health. 2021 Jul 4;18(13):7160. doi: 10.3390/ijerph18137160 (PMC8297147; doi:10.3390/ijerph18137160)
Supplement: Supplementary file 1 [file ijerph-18-07160-s001.zip › ijerph-1247982-supplementary.pdf]

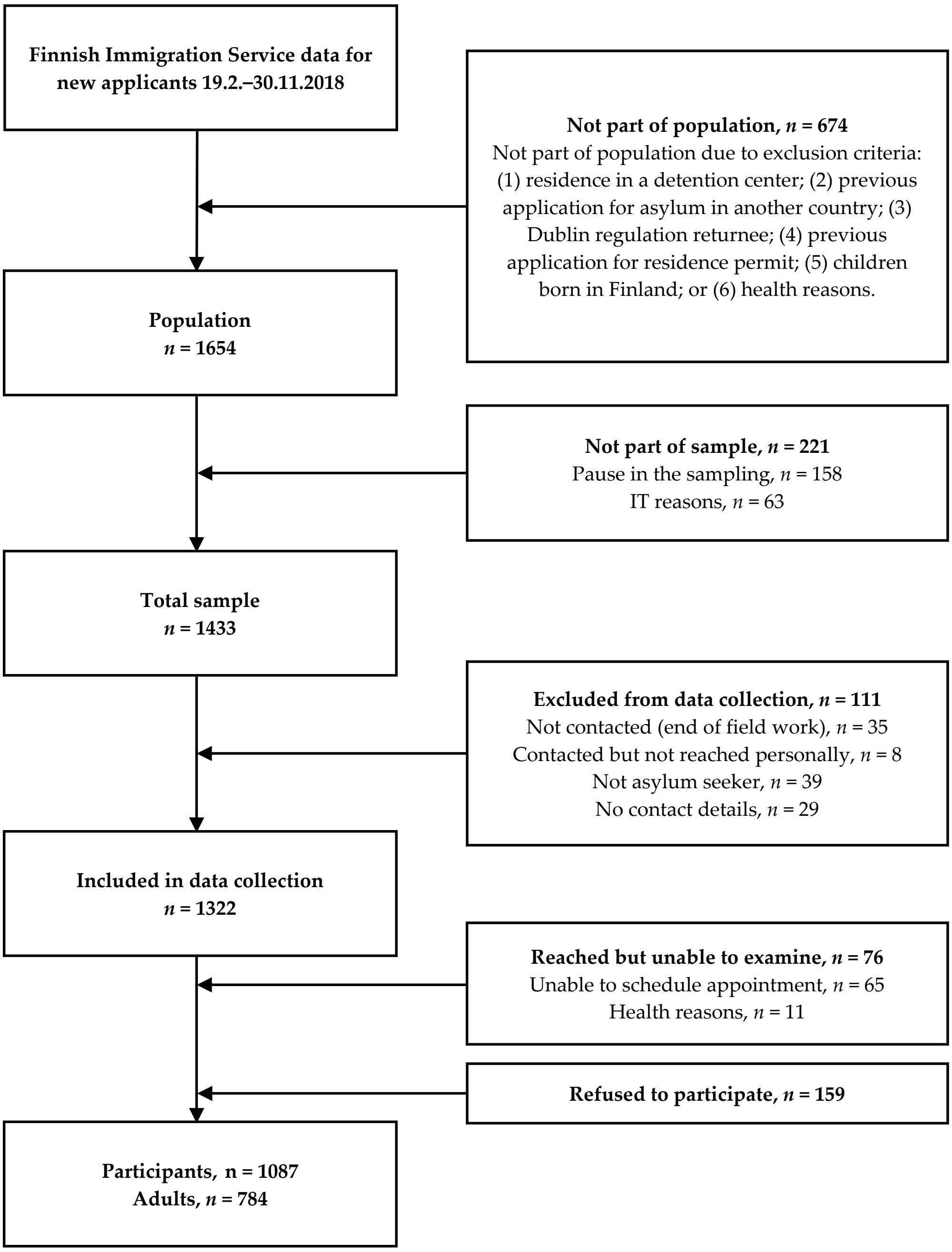

Figure S1. The sampling and data collection process.

Table S1. Age-adjusted prevalence of potentially traumatic events (PTEs) by region of origin before the asylum-seeking journey.

Reported PTEs before the Journey	Region of Origin				Total
	Russia/FSU Total *n*= 229 % (CI)	MENA Total *n*= 332 % (CI)	Africa (excl. NA) Total *n*= 125 % (CI)	Other Total *n*= 83 % (CI)	Total *n*= 769 % (CI)
Combat situations					
Male	20.3 (16.6–24.5)	31.3 (27.6–35.3)	44.0 (37.4–50.9)	35.0 (27.6–43.2)	30.6 (28.2–33.2)
Female	13.7 (10.1–18.3)	28.1 (23.6–33.1)	46.1 (38.0–54.4)	42.8 (32.0–54.3)	28.6 (25.6–31.8)
Total	17.6 (14.9–20.6)	30.0 (27.1–33.1)	45.0 (39.8–50.4)	37.9 (31.6–44.5)	29.8 (27.9–31.8)
Natural disaster					
Male	14.9 (11.7–18.8)	26.2 (22.8–29.9)	12.5 (8.6–17.7)	23.6 (17.3–31.3)	20.4 (18.3–22.7)
Female	11.5 (8.2–15.8)	18.8 (15.2–22.9)	14.7 (9.4–22.1)	32.6 (23.0–43.9)	17.6 (15.2–20.3)
Total	13.5 (11.1–16.3)	23.2 (20.6–25.9)	13.5 (10.2–17.7)	26.9 (21.5–33.2)	19.3 (17.7–21.0)
Seeing violent injury or death					
Male	51.8 (46.8–56.7)	61.1 (57.0–65.0)	75.2 (69.0–80.5)	63.4 (55.4–70.8)	61.0 (58.3–63.6)

Female	33.8 (28.4–39.6)	35.6 (30.7–40.8)	64.1 (55.9–71.6)	57.2 (45.8–67.9)	42.3 (38.9–45.7)
Total	44.5 (40.7–48.3)	50.8 (47.6–53.9)	70.9 (65.9–75.3)	60.5 (54.0–66.8)	53.4 (51.3–55.5)
			Physical harm		
Male	77.9 (73.4–81.7)	47.9 (43.8–52.0)	78.0 (71.6–83.3)	63.2 (55.2–70.6)	62.9 (60.2–65.5)
Female	51.2 (45.3–57.1)	28.8 (24.3–33.8)	58.8 (50.1–67.0)	42.6 (31.8–54.2)	41.9 (38.6–45.3)
Total	67.0 (63.4–70.5)	40.2 (37.1–43.3)	70.2 (65.1–74.9)	54.8 (48.3–61.1)	54.4 (52.3–56.5)
			Physical violence		
Male	69.3 (64.5–73.7)	45.0 (40.9–49.1)	79.1 (73.1–84.0)	58.1 (49.9–65.8)	58.5 (55.9–61.2)
Female	31.6 (26.4–37.3)	24.2 (20.1–28.9)	63.5 (54.9–71.4)	36.3 (26.1–48.0)	34.6 (31.5–37.9)
Total	53.8 (50.1–57.4)	36.5 (33.5–39.6)	73.0 (68.0–77.4)	49.1 (42.6–55.5)	48.8 (46.7–50.9)
			Imprisoned or kidnapped		
Male	42.7 (37.8–47.7)	28.8 (25.2–32.7)	51.0 (44.1–57.8)	30.9 (24.0–38.9	36.2 (33.7–38.9)
Female	9.3 (6.4–13.2)	16.6 (13.1–20.9)	27.7 (20.8–35.8)	28.4 (18.3–41.3)	17.8 (15.2–20.7)
Total	28.8 (25.6–32.3)	23.8 (21.1–26.6)	41.5 (36.4–46.8)	29.5 (23.5–36.3)	28.7 (26.8–30.7)
			Torture		
Male	46.2 (41.3–51.3)	41.8 (37.8–46.0)	57.8 (50.9–64.3)	49.9 (42.0–57.8)	46.4 (43.7–49.1)
Female	7.2 (4.7–10.9)	32.0 (27.4–37.1)	37.2 (29.4–45.8)	37.3 (26.6–49.4)	26.4 (23.5–29.6)
Total	30.0 (26.7–33.5)	37.8 (34.7–41.0)	49.5 (44.2–54.8)	44.5 (38.0–51.1)	38.2 (36.2–40.3)
			Sexual violence		
Male	5.8 (3.9–8.7)	6.3 (4.5–8.7)	15.5 (11.2–21.2)	1.8 [1]	7.0 (5.7–8.5)
Female	14.9 (11.1–19.6)	14.9 (11.7–18.7)	35.1 (27.5–43.5)	24.1 (15.6–35.3)	19.4 (16.8–22.1)
Total	9.5 (7.5–12.0)	9.8 (8.1–11.9)	23.5 (19.3–28.3)	10.6 (7.1–15.5)	12.0 (10.7–13.5)
			Forced or cheated		
Male	37.1 (32.4–42.0)	32.9 (29.2–37.0)	43.8 (37.1–50.6)	38.9 (31.4–47.0)	36.1 (33.5–38.7)
Female	25.9 (21.1–31.3)	27.7 (23.3–32.6)	45.8 (37.5–54.2)	23.5 (15.2–34.5)	30.3 (27.3–33.6)
Total	32.4 (29.0–36.1)	30.8 (27.9–33.9)	44.8 (39.5–50.3)	32.7 (26.9–39.1)	33.7 (31.7–35.8)
			Any PTE		
Male	85.7 (81.8–88.9)	86.4 (83.3–89.0)	94.6 [1]	83.9 (77.3–88.8)	87.3 (85.4–89.0)
Female	71.5 (65.9–76.5)	67.4 (62.4–72.1)	85.3 (77.9–90.5)	82.6 (73.6–89.0)	73.1 (70.0–76.0)
Total	79.9 (76.7–82.8)	78.6 (75.9–81.2)	90.8 (87.1–93.5)	83.0 (77.7–87.3)	81.5 (79.8–83.1)

FSU, former Soviet Union; MENA, Middle-East and North Africa; Africa (excl. NA), Africa excluding North Africa; CI, confidence interval, [1] Confidence interval cannot be calculated due to low or high frequency.

Table S2. Age-adjusted prevalence of potentially traumatic events (PTEs) during the asylum-seeking journey by region of origin.

Reported PTEs during the Journey	Region of Origin<br>Russia/FSU<br>Total n = 229<br>% (CI)	MENA<br>Total n = 332<br>% (CI)	Africa (excl. NA)<br>Total n = 125<br>% (CI)	Other<br>Total n = 83<br>% (CI)	Total<br>Total n = 769<br>% (CI)
			Combat situations		
Male	0.0 [1]	0.5 [1]	5.5 [1]	1.8 [1]	1.3 (0.8–2.0)
Female	0.0 [1]	0.7 [1]	1.7 [1]	0.0 [1]	0.6 [1]
Total	0.0 [1]	0.6 [1]	3.8 (2.3–6.3)	1.1 [1]	1.0 (0.7–1.5)
			Natural disaster		
Male	0.0 [1]	0.7 [1]	0.0 [1]	0.0 [1]	0.3 [1]
Female	0.0 [1]	0.0 [1]	5.2 [1]	0.0 [1]	0.9 [1]
Total	0.0 [1]	0.4 [1]	2.3 [1]	0.0 [1]	0.6 [1]
			Seeing violent injury or death		
Male	0.7 [1]	4.5 (3.1–6.6)	17.6 (12.9–23.5)	3.5 [1]	5.4 (4.3–6.7)
Female	0.0 [1]	1.4 [1]	21.8 (15.4–29.9)	0.0 [1]	4.4 (3.2–6.1)
Total	0.4 [1]	3.3 (2.3–4.6)	19.7 (15.5–24.7)	2.2 [1]	5.0 (4.1–6.0)
			Physical harm		
Male	0.7 [1]	4.2 (2.9–6.1)	22.6 (17.2–29.1)	3.5 [1]	6.1 (4.9–7.5)
Female	1.1 [1]	2.8 [1]	22.1 (15.7–30.2)	2.9 [1]	5.6 (4.2–7.4)
Total	0.9 [1]	3.7 (2.7–5.1)	3.7 (2.7–5.1)	3.3 [1]	5.9 (5.0–7.0)
			Physical violence		
Male	0.7 [1]	3.3 (2.2–5.0)	14.0 (9.9–19.4)	3.6 [1]	4.4 (3.5–5.6)
Female	1.1 [1]	2.1 [1]	21.4 (15.1–29.5)	2.9 [1]	5.3 (3.9–7.1)
Total	0.9 [1]	2.8 (2.0–4.0)	17.1 (13.2–21.9)	3.3 [1]	4.8 (4.0–5.8)
			Imprisoned or kidnapped		
Male	0.0 [1]	3.8 (2.6–5.7)	12.8 (9.0–18.1)	5.3 [1]	4.5 (3.5–5.7)
Female	2.1 [1]	4.6 (2.7–7.6)	23.1 (16.5–31.3)	0.0 [1]	6.5 (5.0–8.5)
Total	0.9 [1]	4.1 (3.0–5.6)	16.9 (13.1–21.6)	3.3 [1]	5.3 (4.4–6.3)
			Torture		

Male	0.0 [1]	4.3 (2.9–6.4)	13.1 (9.1–18.6)	3.5 [1]	4.5 (3.5–5.8)
Female	0.0 [1]	3.5 [1]	19.7 (13.6–27.7)	0.0 [1]	5.0 (3.6–6.8)
Total	0.0 [1]	4.1 (2.9–5.7)	15.9 (12.1–20.7)	2.2 [1]	4.7 (3.8–5.7)
Sexual violence					
Male	0.0 [1]	0.5 [1]	2.8 [1]	1.8 [1]	0.9 [1]
Female	0.0 [1]	2.8 [1]	27.6 (20.7–35.8) [2]	0.0 [1]	6.0 (4.5–7.8)
Total	0.0 [1]	1.4 (0.9–2.4)	13.6 (10.2–17.8)	1.1 [1]	2.9 (2.3–3.8)
Forced or cheated					
Male	0.0 [1]	1.0 [1]	11.9 (8.1–17.2)	3.5 [1]	2.8 (2.0–3.8)
Female	0.0 [1]	4.3 (2.7–6.8)	34.4 (26.8–43.0)	0.0 [1]	7.8 (6.2–9.8)
Total	0.0 [1]	2.3 (1.5–3.4)	21.1 (17.0–25.8)	2.2 [1]	4.8 (4.0–5.8)
Any PTE					
Male	1.5 [1]	13.3 (10.7–16.3)	28.0 (22.2–34.6)	5.3 [1]	11.6 (10.0–13.4)
Female	3.2 [1]	8.9 (6.3–12.4)	43.1 (35.0–51.5)	2.9 [1]	12.6 (10.5–15.0)
Total	2.2 (1.3–3.7)	11.6 (9.6–13.8)	34.5 (29.5–39.8)	4.4 [1]	12.0 (10.7–13.4)

FSU, former Soviet Union; MENA, Middle-East and North Africa; Africa (excl. NA), Africa excluding North Africa; CI, confidence interval, [1] Confidence interval cannot be calculated due to low or high frequency, [2] Unstandardized scores are reported due to small cell sizes in other categories.
